# Adenosine Receptor Stimulation Improves Glucocorticoid-Induced Osteoporosis in a Rat Model

**DOI:** 10.3389/fphar.2017.00558

**Published:** 2017-09-05

**Authors:** Gabriele Pizzino, Natasha Irrera, Federica Galfo, Giacomo Oteri, Marco Atteritano, Giovanni Pallio, Federica Mannino, Angelica D’Amore, Enrica Pellegrino, Federica Aliquò, Giuseppe P. Anastasi, Giuseppina Cutroneo, Francesco Squadrito, Domenica Altavilla, Alessandra Bitto

**Affiliations:** ^1^Department of Clinical and Experimental Medicine, University of Messina Messina, Italy; ^2^Department of Biomedical Sciences, Dentistry and Morphological and Functional Images, University of Messina Messina, Italy

**Keywords:** osteoporosis, adenosine, PDRN, glucocorticoids, rats

## Abstract

Glucocorticoid-induced osteoporosis (GIO) is a secondary cause of bone loss. Bisphosphonates approved for GIO, might induce jaw osteonecrosis; thus additional therapeutics are required. Adenosine receptor agonists are positive regulators of bone remodeling, thus the efficacy of adenosine receptor stimulation for treating GIO was tested. In a preventive study GIO was induced in Sprague-Dawley rats by methylprednisolone (MP) for 60 days. Animals were randomly assigned to receive polydeoxyribonucleotide (PDRN), an adenosine A2 receptor agonist, or PDRN and DMPX (3,7-dimethyl-1-propargylxanthine, an A_2_ antagonist), or vehicle (0.9% NaCl). Another set of animals was used for a treatment study, following the 60 days of MP-induction rats were randomized to receive (for additional 60 days) PDRN, or PDRN and DMPX (an adenosine A2 receptor antagonist), or zoledronate (as control for gold standard treatment), or vehicle. Control animals were administered with vehicle for either 60 or 120 days. Femurs were analyzed after treatments for histology, imaging, and breaking strength analysis. MP treatment induced severe bone loss, the concomitant use of PDRN prevented the developing of osteoporosis. In rats treated for 120 days, PDRN restored bone architecture and bone strength; increased b-ALP, osteocalcin, osteoprotegerin and stimulated the Wnt canonical and non-canonical pathway. Zoledronate reduced bone resorption and ameliorated the histological features, without significant effects on bone formation. Our results suggest that adenosine receptor stimulation might be useful for preventing and treating GIO.

## Introduction

Glucocorticoid (GC)-induced osteoporosis (GIO) is the most important secondary cause of bone loss (Van Staa et al., 2002). GC have a direct negative effect on bone cells, can interfere with the body’s handling of calcium and affect levels of sex hormones, leading to bone loss and eventually fracture risk (Van Staa et al., 2000).

To prevent and treat GIO the FDA has approved several drugs, the antiresorptive bisphosphonates (alendronate, risedronate, and zoledronate); and the recombinant parathyroid hormone, teriparatide. None of these drugs has enough safety data available to recommend their use in pregnant or breastfeeding women. Bisphosphonates may also act synergistically with glucocorticoids in inducing osteonecrosis of the jaw (Rutkowski, 2011; Khan et al., 2015), and teriparatide has lots of compliance issues (Bultink et al., 2013); thus there is need of additional therapeutic strategies. Recent research has implicated adenosine as an important regulator in bone remodeling, especially through the A_2A_ receptor (Mediero et al., 2013, 2015; He et al., 2013). Adenosine receptors are a family of 4 G-protein coupled receptors expressed in several tissues and cell types. They play a pivotal role in processes such as embryogenesis, cell differentiation, and tissue remodeling. Some of them (i.e., A_2A_ and A_1_) have been already described as positive regulators of bone remodeling (Mediero et al., 2015). Agonists of A_2A_ receptor could play a beneficial role in preventing the onset, or improving an already existing osteoporotic process.

The polydeoxyribonucleotide (PDRN) is a well characterized A_2A_ selective agonist (Thellung et al., 1999) holding a mixture of deoxyribonucleotides polymers ranging between 50 and 2000 bp (Thellung et al., 1999), it has osteoblast-promoting properties (Guizzardi et al., 2003), and prevents cartilage loss and inflammation in an experimental model of rheumatoid arthritis (Bitto et al., 2011). Given PDRN pharmacological properties, we hypothesized that its administration could prevent the onset of GIO, and/or ameliorate an already established osteoporosis. To assess this hypothesis, we designed an *in vivo* experiment on a rat model of GIO. PDRN was administered in prevention and treatment, while DMPX (an A_2A_ selective antagonist) was employed as tool to assess the involvement of the A_2A_ receptor subtype. Finally, we decided to administer zoledronic acid as benchmark for anti-osteoporotic efficacy.

## Materials and Methods

### Animal Model

All procedures were evaluated and approved by the Ethics Committee of Messina University and complied with the ARRIVE guidelines (Kilkenny et al., 2010). A total of 78, 5-month-old female Sprague–Dawley rats (Charles River, Calco, Italy) weighing 250–275 g were used. Animals were housed in plastic cages (*n* = 3–4/cage), in our Animal Facility, maintained under controlled environmental conditions (12 h light/darkness cycle, temperature 24°C), and provided with standard food and water *ad libitum*. The administration of methylprednisolone (Sigma–Aldrich, Milan, Italy) at the dose of 30 mg/kg is equivalent to a human dose of 7.5 mg, commonly prescribed for rheumatic disease. At the time of killing the samples were coded and all the results have been obtained blinded.

### Prevention Study

All groups belonging to this study have been indicated with a p (prevention). GIO was induced by daily s.c. injections of 30 mg/kg of methylprednisolone (hereinafter pMP; *n* = 21) for 60 days, as previously described (Bitto et al., 2009b). Of the pMP animals, 7 were randomly assigned to concomitantly receive PDRN (hereinafter pMP+PDRN; 8 mg/kg i.p.), 7 were randomly assigned to concomitantly receive PDRN and 10 mg/kg of DMPX (adenosine A2 receptor agonist; Sigma–Aldrich, Milan, Italy) (hereinafter pMP+PDRN+DMPX), and the other 7 saline solution (in a volume of 200 μl i.p.). Control sham animals received saline (pCTRL; *n* = 5). All animals were euthanized at the end of the 60 days of treatment under general anesthesia.

### Treatment Study

All groups belonging to this study have been indicated with a t (treatment). In the second experimental setting GIO was induced by daily s.c. injections of 30 mg/kg of methylprednisolone (hereinafter tMP; *n* = 42) for 60 days. After 60 days the tMP animals were randomly assigned to receive PDRN (8 mg/kg i.p. daily; *n* = 14, hereinafter tMP+PDRN), or PDRN and 10 mg/kg of DMPX (hereinafter tMP+PDRN+DMPX; *n* = 7), or zoledronic acid (Sigma–Aldrich, Milan, Italy) (7.5 μg/kg i.p. once a week; *n* = 7, hereinafter tMP+Z), and the other 14 saline solution (in a volume of 200 μl i.p.; hereinafter tMP+S), to evaluate the spontaneous recovery from GIO. Control sham animals received saline (tCTRL; *n* = 10) for all the 120 days. At the end of the 120 days all animals were euthanized under general anesthesia, femurs were removed and tested. Animals from the tCTRL (*n* = 5), tMP+S (*n* = 7), and tMP+PDRN (*n* = 7) were also used for measuring bone mineral density and microCT scan; and to obtain primary osteoblasts for quantitative polymerase chain reaction (qPCR) analysis. Experimental procedures and drug dosages were chosen in agreement to previous studies (Hornby et al., 2003; Bitto et al., 2009a,b, 2013).

### Bone Breaking Strength

After killing the lower limbs were disarticulated and one of the femurs used for evaluating the maximum tolerated load (breaking strength), using a calibrated tensometer (Sans, China) as previously described (Bitto et al., 2009a,b).

### Osteoblast Culture

Primary osteoblasts were obtained from the femurs of animals belonging to the tCTRL, tMP, and tMP+PDRN groups. Femurs were kept in cold PBS until use, epiphyses were removed and bone marrow was flushed with PBS. Diaphysis were cut into little pieces of 1–2 mm, washed several time with PBS and incubated in a 4 ml collagenase II (2 mg/ml in DMEM) solution at 37°C on the shaker to remove all soft tissue for 2 h. Bone pieces were rinsed with culture medium (DMEM added with 10% FBS, 100 U/ml penicillin and 50 μg/ml streptomycin sulfate) (Sigma–Aldrich, Milan, Italy) and transferred into flasks at a density of about 20–30 fragments per flask. Cells were kept under standard conditions (37°C and 5% CO_2_ atmosphere) and medium was replaced every 3 days; cell migration was observed from bone pieces starting at day 3 and cells were used for the qPCR experiment at day 15.

### PCR Assays

Total RNA was extracted from osteoblasts at day 15 using Trizol LS reagent (Invitrogen, Carlsbad, CA, United States), quantified with a spectrophotometer (NanoDrop Lite, Thermo Fisher) and 400 ng was reverse transcribed using the Superscript VILO kit (Invitrogen) in a volume of 50 μl. The qPCR analysis was performed as follow: 1 μl of cDNA was added to the EvaGreen qPCR Master Mix (Biotium Inc., Fremont, CA, United States), in a total volume of 20 μl per well. Samples were loaded in duplicate, GADPH was used as housekeeping gene; the reaction was performed using the 2-step thermal protocol suggested by the manufacturer. We tested three different primer concentrations (100, 300, and 900 nM) and 300 nM was selected and used to perform the analysis. Target genes were Wnt10b, Wnt5a, beta catenin, Secreted Frizzled-Related Protein 1 (sFrp1), and Secreted Frizzled-Related Protein 2 (sFrp2). Primers used for both target and reference genes are listed below:

Gadph- Fw: 5′-GTCAAGGCTGAGAATGGGAA-3′ Rv: 5′-ATACTCAGCACCAGCATCAC-3′; Wnt5a-Fw: 5′-CCATGAAGAAGCCCATTGGAATA-3′ Rv: 5′-GGCCAAAGCCATTAGGAAGAA-3′; Wnt10b-Fw: 5′-CAGAACCACCCGTGAGTTAG-3′ Rv: 5′-GGGAGGGAGTGATCCAGATA-3′; Beta-Catenin-Fw: 5′-CGGCACCTTCCTATTTCTTCT-3′ Rv: 5′-TCTGGAAATTAACTTCAGGCAAAC-3′; sFrp1-Fw: 5′-GGCTACAAGAAGATGGTGCT-3′ Rv: 5′-GCCCATGTGGCAGTTCT-3′; sFrp2-Fw: 5′-AGCCCGACTTCTCCTACA-3′ Rv: 5′-CGCATGTTCTGGTACTCGAT-3′.

Results were calculated using the 2^-ΔΔC_*t*_^ method, and expressed as *n*-fold increase in gene expression using as calibrator the results from osteoblasts obtained of the tCTRL group.

### Biochemical Analysis

Blood collection was performed by cardiac puncture, after centrifugation and serum stored at -20°C. Stored samples were used to determine the levels of Bone-Alkaline Phosphatase (BALP) and Cross linked C-telopeptide of Type I collagen (CTX-I) using rat-specific commercially available ELISA kits (Elabscience, Wuhan, China).

### Radiography

Before formalin fixation the images from excised femurs have been acquired with a digital radiographic system XIOS type D3495 (Sirona Dental Systems GmbH, Bensheim, Germany) Sensor active surface: 20 mm × 30 mm, with a 7 mA 60 kW exposure for 0.05 s.

### Femur BMD Measurement

The left femur from each rat was harvested with a trace amount of surrounding soft tissues and immersed in 0.9% saline solution. BMD levels of the femora were measured with a dual-energy X-ray absorptiometer (DEXA; LUNAR Radiation, Madison, WI, United States) using the small-animal program.

### *Ex Vivo* Microcomputed Tomography

The excised femurs and spines were maintained in 10% neutral buffered formalin at room temperature until micro-CT scanning. All bones were scanned parallel to the transverse plane by using micro-CT. The images of each femur and spine were obtained using a Skyscan 1176 micro-CT device (Bruker, Konich, Belgium). The scanning parameters were set at a voxel resolution of 9 μm and aluminum filter; tomographic image reconstruction was performed using NRecon software (Bruker). To determine the trabecular bone microarchitecture at femoral and spine level, the bone volume fraction (bone volume/total volume, BV/TV, unit = %) and trabecular bone thickness (TbTh, unit = mm) were evaluated.

### Histology

Tissue was collected disarticulating the leg at the hip and knee. Femurs were removed, and processed as previously described (Bitto et al., 2009a,b). Femoral heads (area comprised between hip joint cartilage and metaphyseal cartilage) were used to judge the quality of cartilage, bone, and trabecular density according to the scores previously published (Hornby et al., 2003; Bitto et al., 2009a,b, 2013). Briefly, the score take in account for the % of trabecular bone area (TBA): 0 (90–100% TBA), 1 (60–90% TBA), 2 (30–60% TBA), and 3 (0–30% TBA) of the femur head. The score were assigned by 2 observers blinded to treatments. Osteoblasts were identified according to their histological characteristics by two pathologists. Counting was performed along the bone surface at 40× magnification using an eyepiece micrometer. The bone length was measured using Image J program^1^. Both the cell count and measurement processes were repeated three times and the average was calculated.

### Immunohistochemistry

Immunostaining for osteocalcin, osteoprotegerin (OPG), Wnt5a, and Wnt10b (all from Abcam, Cambridge, United Kingdom) was performed on 5-μm-thick paraffin-embedded horizontal bone sections. The Vectastain ABC Elite kit (Vector Laboratories, INC., Burlingame, CA, United States) was used to develop the immunoenzymatic reaction. Counterstain was performed with hematoxylin, and then the slides were observed by two observers blinded to treatments. Counting of positive cells was performed along the bone surface at 40× magnification using an eyepiece micrometer.

### Statistical Analysis

All quantitative data are expressed as mean ± SD or mean ± SEM for each group, and compared by using one-way or two-way ANOVA for non-parametric variables, with Tukey post-test for intergroup comparisons. Statistical significance was set at *p* < 0.05. Graph were drawn using GraphPad Prism software version 5.0 for Windows (GraphPad Software Inc., La Jolla, CA, United States).

## Results

### Adenosine Receptor Activation Improves Bone Morphology and Strength

**FIGURE 1:**
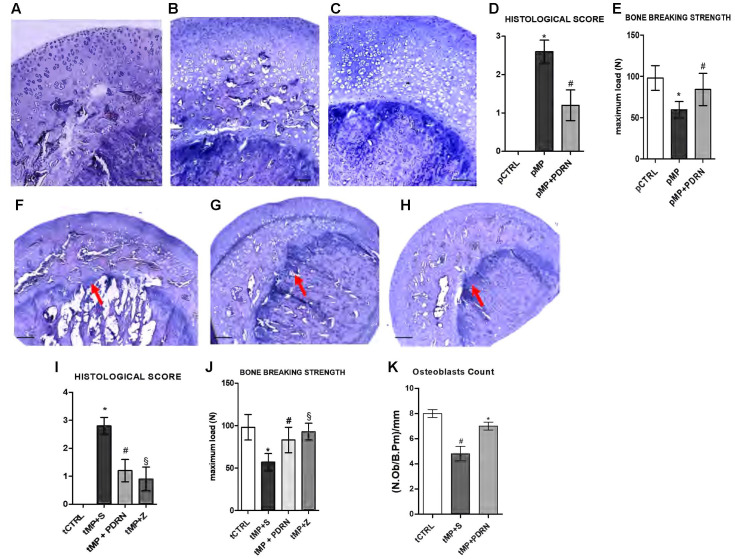
Bone histology – prevention and treatment study. **(A–C)** Representative H&E staining of femur heads, scale bar 50 μm. **(A)** pCTRL, **(B)** pMP, **(C)** pMP-PDRN. The graph in **(D)** represents the histological score; the graph in **(E)** the breaking strength for each group. ^∗^*p* < 0.001 pMP vs. pCTRL; ^#^*p* < 0.01 pMP+PDRN vs. pMP. Data are expressed as means and SD. **(F–H)** Representative H&E staining of femur heads, scale bar 100 μm. **(A)** tMP+S, **(B)** tMP+PDRN, **(C)** tMP+Z. The arrows point at the metaphyseal cartilage. The graph in **(I)** represents the histological score; the graph in **(J)** the breaking strength for each group. ^∗^*p* < 0.001 tMP+S vs. tCTRL; ^#^*p* < 0.01 tMP+PDRN vs. tMP+S; ^§^*p* < 0.01 tMP+Z vs. tMP+S. **(K)** Osteoblasts count, expressed as number of osteoblasts per mm of bone perimeter. ^∗^*p* < 0.05 vs. tMP+S; ^#^*p* < 0.05 vs. tCTRL. Data are expressed as means and SD.

The histological analysis revealed osteoporotic lesions in pMP compared to pCTRL animals (**Figures 1A,B**). The concomitant administration of PDRN prevented the occurrence of osteoporosis in treated animals (**Figure 1C**). The histological score (**Figure 1D**) representing the mean values for each group confirmed the protective role of PDRN. After 60 days of MP administration the resistance-to-fracture of the femurs was significantly lower compared to controls (**Figure 1E**, *p* < 0.001 pMP vs. pCTRL). PDRN restored the impaired bone strength (*p* < 0.01 pMP+PDRN vs. pMP). When DMPX was co-administered with MP and PDRN, the improvements in both the histological features and the resistance-to-fracture were abrogated (data not shown).

In the treatment study tMP animals were not able to recover from osteoporosis when administered with saline solution for additional 60 days (**Figure 1F**), the deterioration of the metaphyseal plate and the reduction in osteoblasts were more evident. In this context of established GIO, the administration of the A_2A_ agonist promoted bone regeneration (**Figure 1G**) as evidenced by the restored metaphyseal cartilage and the increased presence of osteoblasts. Treatment with zoledronate was able to improve bone morphology; however, did not markedly increase osteoblasts (**Figure 1H**). The histological score in **Figure 1I** confirmed the efficacy of PDRN in improving bone quality (*p* < 0.01 tMP+PDRN vs. tMP+S). Also the breaking strength analysis indicated that both treatments were able to increase the maximum tolerated force of femurs (**Figure 1J**). Antagonizing the A_2A_, the effects of PDRN were abolished (data not shown). Osteoblasts number was significantly reduced in tMP+S rats compared to controls, while PDRN significantly improved their number (**Figure 1K**).

### Radiographic and Densitometric Bone Evaluation

**FIGURE 2:**
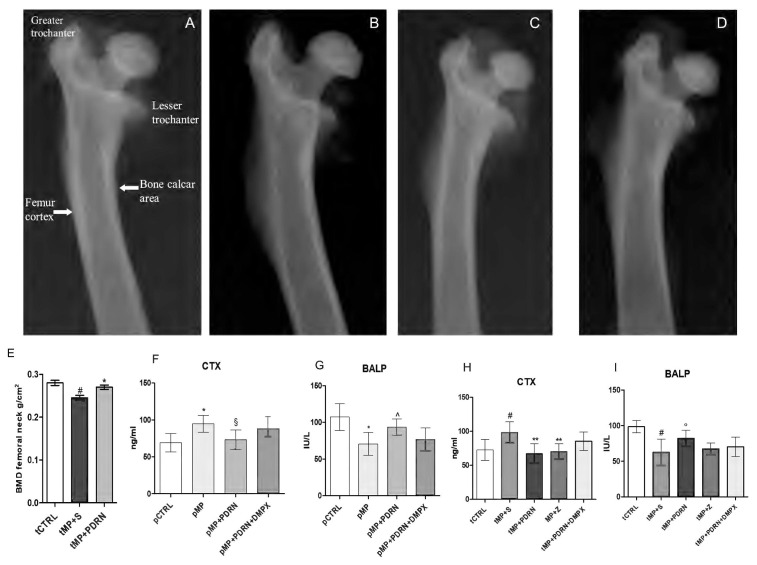
Bone x-rays and markers. **(A–D)** Representative X-ray images obtained from the several study group. **(A)** tCTRL, **(B)** tMP+S, **(C)** tMP+PDRN, **(D)** tMP+Z. **(E)** BMD at femoral neck expressed as g/cm^2^ in the several groups. ^∗^*p* < 0.05 vs. tMP+S; ^#^p < 0.05 vs. tCTRL. Data are expressed as means and SD. Bone resorption (CTX; **F,H**) and formation (BALP; **G,I**) markers evaluated in the several study groups. ^∗^*p* < 0.005 vs. pCTRL; ^§^*p* < 0.01 vs. pMP; ^∧^*p* = 0.02 vs. pMP; ^#^*p* < 0.005 vs. tCTRL; ^∗∗^*p* < 0.01 vs. tMP+S; °*p* < 0.05 vs. tMP+S. Data are expressed as means and SD.

In the treatment study, the X-ray films obtained from tMP+S animals demonstrate a reduced intensity in the diaphysis, metaphysis, and epiphysis compared to controls, suggestive of reduced calcium presence (**Figures 2A,B**). Additionally, a thinning of the cortical bone is also evident in tMP+S compared to tCTRL. Both treatments improved these hallmarks (**Figures 2C,D**) of bone rarefaction and the effect was more evident with the administration of the adenosine A_2A_ agonist (**Figure 2C**).

Bone densitometry (**Figure 2E**) of tCTRL animals showed a BMD at femoral neck of 0.0272 ± 0.004, as expected tMP+S group demonstrated a reduced bone mineral content (0.241 ± 0.011; *p* < 0.05 vs. tCTRL), while the group treated with PDRN had a significant improvement in BMD values (0.262 ± 0.007; *p* < 0.05 vs. tMP+S).

### Biochemical Markers

CTX assay showed that pMP animals had increased levels compared to pCTRL (*p* < 0.005), indicating an increased bone resorption. The pMP+PDRN group showed a significant reduction of this marker (*p* < 0.01), suggesting an anti-resorptive activity of PDRN (**Figure 2F**) when administered concomitantly with corticosteroids. The bone specific alkaline phosphatase (**Figure 2G**) was also investigated to determine the bone remodeling *status*; pMP animals demonstrated reduced levels of this protein when compared to pCTRL rats (*p* < 0.005), while the administration of PDRN markedly increased BALP serum content (*p* = 0.02), abrogated by DMPX co-administration.

The assay of CTX in the treatment study showed that tMP+S animals had a further increase in the circulating levels of this marker as compared to the tCTRL group (*p* < 0.005), indicating a persistency of the bone resorptive effect of glucocorticoids. The tMP+PDRN group instead, showed a significant reduction of this marker (*p* < 0.01), suggesting that PDRN (**Figure 2H**) is also able to revert an established bone loss condition. Zoledronate successfully reduced CTX levels (*p* < 0.01 vs. tMP+S). BALP levels (**Figure 2I**) also indicated that MP+S animals did not undergo to spontaneous recovery of their altered bone metabolism; in fact, BALP levels were still reduced compared to rats in the tCTRL group (*p* < 0.005). The administration of PDRN was able to increase BALP serum content (*p* < 0.05), suggesting a bone formation effect. Zoledronic acid treatment did not affect BALP levels (**Figure 2I**). Finally, even in this setting DMPX was able to negatively affect the pharmacological action of PDRN.

### *Ex Vivo* Microcomputed Tomography Evaluations

**FIGURE 3:**
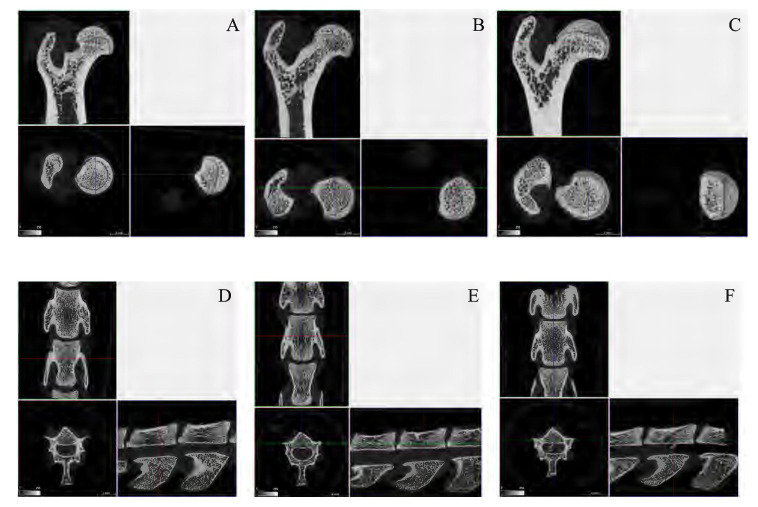
Bone micro-CT. **(A–C)** Representative micro-CT images obtained from femurs of the several study group. **(A)** tCTRL, **(B)** tMP+S, **(C)** tMP+PDRN. **(D–F)** Representative micro-CT images obtained from spines of the several study group. **(A)** tCTRL, **(B)** tMP+S, **(C)** tMP+PDRN.

The analysis performed by micro-CT (**Figures 3A–F**) highlighted a significant amelioration in several parameters, both at femoral and spinal level. The average Bv/Tv (Bone volume/Tissue volume) ratio was impaired in MP+S rat femurs (47.88 ± 2.58 vs. 72.41 ± 1.95) and spines (32.75 ± 5.3 vs. 42.42 ± 3.43) compared to controls; when treated with PDRN, rat had an improved average Bv/Tv ratio at both skeletal sites (femurs 73.63 ± 3.93, *p* < 0.001 vs. tMP+S; spines 52.76 ± 5.83, *p* < 0.001 vs. tMP+S). The same is true even considering trabecular thickness, at both femoral (0.138 ± 0.016 vs. 0.102 ± 0.006, tMP+PDRN vs. tMP+S; *p* < 0.05) and spinal site (0.098 ± 0.004 vs. 0.086 ± 0.006, tMP+PDRN vs. tMP+S; *p* < 0.05). As reference, tCTRL rats showed a femoral trabecular thickness of 0.162 ± 0.004 and spinal trabecular thickness of 0.096 ± 0.003.

### Adenosine Receptor Activation Improves Bone Formation

To better understand the PDRN ability to restore bone formation following corticosteroid treatment, we analyzed bone specific markers in the femurs obtained from the treatment study.

**FIGURE 4:**
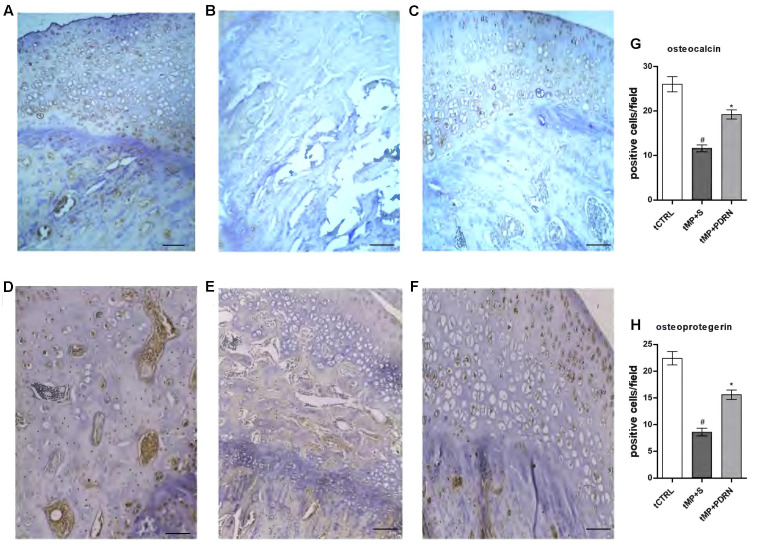
Osteocalcin and osteoprotegerin immunostaining. **(A–C)** Representative osteocalcin immunostaining of femoral head sections, scale bar 10 μm. Haematoxylin counterstain. **(A)** tCTRL, **(B)** tMP+S; **(C)** tMP+PDRN. **(D,E)** Representative osteoprotegerin immunostaining of femoral head sections, scale bar 10 μm. Haematoxylin counterstain. **(D)** tCTRL, **(E)** tMP+S; **(F)** tMP+PDRN. **(G,H)** The graphs represent the number of positive cells for osteocalcin **(G)** and osteoprotegerin **(H)**, per high power field. ^∗^*p* < 0.05 vs. tMP+S; ^#^*p* < 0.05 vs. tCTRL. Data are expressed as means and SD.

As shown in (**Figures 4A–C**) osteocalcin (a marker of bone formation) is expressed in tCTRL animals and markedly reduced in the tMP+S group. In the tMP+PDRN group the expression of osteocalcin was almost restored, as shown in **Figure 4C**.

Another bone formation marker is osteoprotegerin (**Figures 4D–F**), normally expressed in bones obtained by the tCTRL group and markedly reduced in the tMP+S group. In the tMP+PDRN group OPG expression was substantially restored (**Figure 4F**). When quantified, these results proved to be statistically significant. In fact, both osteocalcin and OPG levels were significantly under-expressed in tissue sections of tMP+S animals, compared to tCTRL (*p* < 0.05). PDRN significantly improved these molecular markers (*p* < 0.05 vs. tMP+S) (**Figures 4G,H**). These observations suggest that A_2A_ receptor stimulation is important to stimulate bone formation. Zoledronic acid treated animals did not show an increase in either osteocalcin and osteoprotegerin expression (results not shown).

**FIGURE 5:**
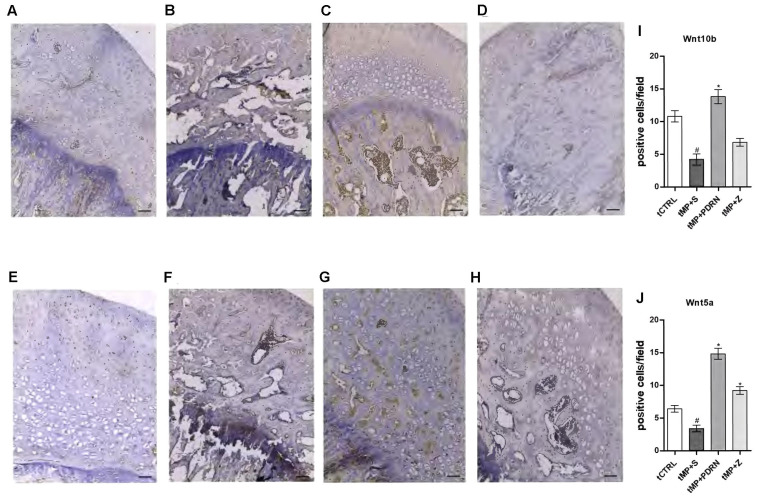
Wnt immunostaining. **(A–D)** Representative Wnt10b immunostaining of femoral head sections, scale bar 20 μm. Haematoxylin counterstain. **(A)** tCTRL, **(B)** tMP+S; **(C)** tMP+PDRN; **(D)**: tMP+Z. **(E–H)** Representative Wnt5a immunostaining of femoral head sections, scale bar 20 μm. Haematoxylin counterstain. **(E)** tCTRL, **(F)** tMP+S; **(G)** tMP+PDRN; **(H)**: tMP+Z. **(I,J)** The graphs represent the number of positive cells for Wnt10b **(I)** and Wnt5a **(J)**, per high power field. ^∗^*p* < 0.05 vs. tMP+S; ^#^*p* < 0.05 vs. tCTRL. Data are expressed as means and SD.

The canonical (Wnt10b) and non-canonical (Wnt5a) activation of Wnt signaling were also investigated by immunostaining as shown in **Figure 5**. The obtained results showed that the canonical Wnt10b has a basal expression in the normal femur head (**Figure 5A**), while tMP+S treatment almost abolished its expression (**Figure 5B**). In the treated groups only the stimulation of the adenosine receptor was successful in increasing Wnt10b, while the zoledronic acid did not show signs of improvement of this canonical pathway (**Figures 5C,D**). The non-canonical pathway investigated by the expression of Wnt5a revealed, as expected, a very low presence in the normal bone (**Figure 5E**) as well as in the tMP+S femurs (**Figure 5F**). In the tMP+PDRN group a strong induction of Wnt5a expression was observed (**Figure 5G**), while the tMP+Z group demonstrated only a very moderate staining for this marker (**Figure 5H**). The quantification showed that Wnt10b was significantly augmented by PDRN treatment (*p* < 0.05 tMP+PDRN vs. tMP+S) while it was under-regulated by methylprednisolone administration (*p* < 0.05 tCTRL vs. tMP+S) (**Figure 5I**). The same was true when Wnt5a was quantified, but in this case we reported an increase even when animals were treated with zoledronate (**Figure 5J**). The activation of both the canonical and non-canonical Wnt signaling pathway is a further indicator of the pro-regenerative activity achieved by adenosine A_2A_ receptor stimulation, confirmed by the fact that the DMPX-mediated blockade of this receptor significantly impaired the anti-osteoporotic effects observed after PDRN administration (not shown).

### Gene Expression Analysis

**FIGURE 6:**
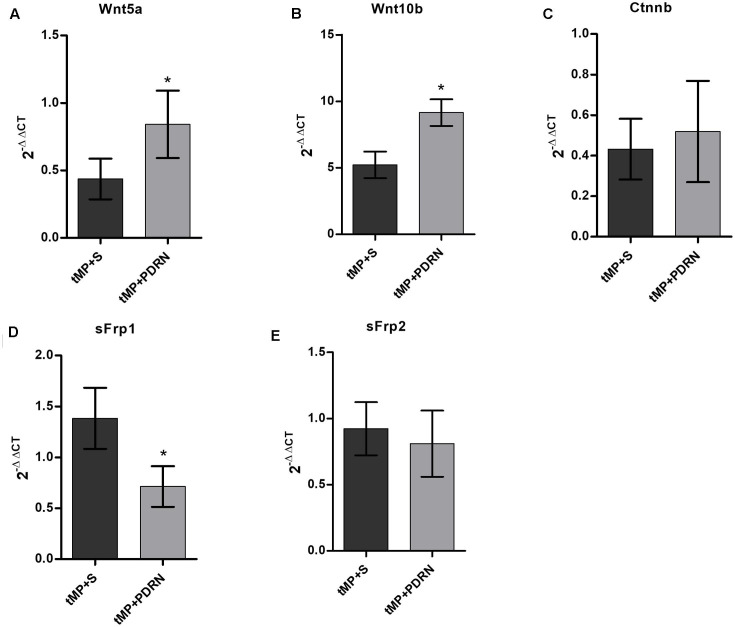
quantitative polymerase chain (qPCR) reaction on primary osteoblasts. qPCR results obtained from primary osteoblasts. **(A)** Wnt5a. **(B)** Wnt10b. **(C)** Ctnnb. **(D)** sFrp1. **(E)** sFrp2. ^∗^*p* < 0.05. Data are expressed as means and SD.

Wnt5a showed a down-regulation in tMP+S rats compared to controls (assumed as reference value of 1; not showed in graph); it is worth to note that PDRN treatment seems to partially revert this phenomenon, determining a 100% increase in Wnt5a expression comparing tMP+S and PDRN-treated animals (*p* < 0.05; **Figure 6A**).

Wnt10b were strongly up-regulated in both tMP+S and tMP+PDRN animals (five and ninefolds the reference, respectively); this up-regulation were significantly (*p* < 0.05) more robust in animals treated with PDRN than in those undergoing to spontaneous recovery (**Figure 6B**).

When comes to β-catenin, we observed a similar but not significant degree of down-regulation in both tMP+S and tMP+PDRN rats (**Figure 6C**).

We analyzed then two Wnt/β-catenin regulator genes, sFrp1 and sFrp2. The first, sFrp1, was up-regulated in tMP+S and significantly down-regulated in PDRN-treated rats (*p* < 0.05; **Figure 6D**).

The second, sFrp2, showed any significant up-/down-regulation compare to the reference value from control rats, both in tMP+S and PDRN-treated group (**Figure 6E**).

## Discussion

Adenosine is generated intracellularly and extracellularly from the catabolism of adenine nucleotides and regulates a variety of physiological processes via interaction with specific G-protein-coupled receptors. Four subtypes are currently recognized: A_1_, A_2A_, A_2B_, and A_3_ receptors, which are virtually present in every tissue. Deletion or blockade of A_1_ leads to increased bone density and prevents ovariectomy-induced bone loss without affecting bone formation (Kara et al., 2010), and A_1_ activation is required for osteoclast maturation and function *in vitro* (He et al., 2013).

Furthermore, Mediero et al. (2015) demonstrated *in vivo* that A_2A_ are involved in new bone formation, both when stimulated by a selective agonist and when the adenosine tissue levels are increased; they used the CGS21680 as A_2A_ agonist, or dipyridamole to increase adenosine levels. The results were abrogated when an A_2A_ antagonist is used, or when this receptor is deleted. PDRN is used in clinical practice as a tissue repair and stimulating agent (Squadrito et al., 2014), and is extracted from the sperm of trout bred for feeding purposes. It has been suggested that, after entering into cells, PDRN is cleaved by active cell membrane enzymes, providing a source for the synthesis of purine and pyrimidine deoxynucleosides and deoxyribonucleotides, increasing the proliferation rate in several tissues (Sini et al., 1999; Thellung et al., 1999; Guizzardi et al., 2003, 2007).

The currently used drugs for treating GIO largely focuses on decreasing bone resorption; here we provide evidence that PDRN produced a marked improvement in bone formation. Staining for osteocalcin, a marker of bone calcification and osteoblast function, revealed a moderate increase in femurs treated with PDRN, indicating a stimulation of bone formation, since it binds to hydroxyapatite crystals. On the contrary, the serum increase in osteocalcin would account for increased osteoclast activity. As confirmation, we determined also OPG expression in femur sections; in fact, osteocalcin released from bone by osteoclast erosion is able to stimulate insulin release, this latter binds to its receptor on osteoblasts damping OPG production (Karsenty and Ferron, 2012). Thus, it is not surprising that the augmented presence of osteocalcin in bone paralleled the increase in osteoprotegerin and BALP, with a decreased release of CTX. To better understand the mechanism(s) by which adenosine receptor stimulation increased bone formation and restored bone architecture following glucocorticoid treatment, we investigated the canonical and non-canonical Wnt activation pathway. Wnt signaling is vital for osteoblast differentiation and bone mass maintenance (Krishnan et al., 2006); in fact, mice overexpressing Wnt10b in bone marrow maintain bone mass during aging while the expression of Wnt5a is decreased by aging (Rauner et al., 2008). Furthermore, Wnt5a seems to play a crucial role in osteoblastogenesis: it has been observed that Wnt5a non-canonical signaling contributes to a proper bone formation (Okamoto et al., 2014). Wnt5a^+/-^ mice suffer of reduced bone mass, increased adipogenesis, and reduced osteoblastogenesis (Maeda et al., 2012). Wnt10b is a canonical Wnt/β-catenin activator required to bone maintenance, in fact, Wnt10b-null mice showed an age-dependent bone loss (Stevens et al., 2010). sFrp1 and sFrp2 are known to be relevant co-regulators of Wnt/β-catenin pathway, involved in bone formation. sFrp1 acts as a negative regulator of bone formation. In fact, sFrp1^-/-^ mice showed increased trabecular bone mineral density, volume, and mineral apposition rate compared with wild-type littermates. Loss of sFRP1 reduces both osteoblast and osteocyte apoptosis *in vivo*, while *in vitro* it seems that deleting sFrp1 can enhance osteoblasts proliferation and differentiation. sFrp1 deletion may cause an increased *in vitro* osteoclastogenesis, although *in vivo* any change in bone resorption has been detected (Bodine et al., 2004). Even sFrp2 has been described as a negative regulator of Wnt pathway; little is known about the exact role of this inhibitor in bone microenvironment, but there are evidences of its involvement in multiple myeloma and ameloblastoma, where impairs osteoblast differentiation and bone formation, probably suppressing bone morphogenetic protein-2 activity (Oshima et al., 2005; Sathi et al., 2008). In addition, glucocorticoids are known to transcriptionally stimulate the expression of Wnt inhibitors as Dickkopf protein (Dkk 1–4) and secreted Frizzled-Related Proteins (Sfrp) families, which result in loss of β-catenin to ROS-activated FoxO transcription factors (Guañabens et al., 2014). Dkk-1, -2, and -4 function as antagonists of canonical Wnt signaling by either binding to LRP-5/6, or to Kremen-1. sFRP-1-5 bind to Wnt proteins through their N-terminal cysteine-rich domains (CRDs), homologous to the CRDs found in the Frizzled family of Wnt receptors. Nevertheless, sFRPs may also enhance Wnt signaling by stabilizing or transporting Wnt ligands. In addition to binding to Wnt proteins, sFRPs can also inhibit Wnt signaling pathways by binding directly to Frizzled receptors (Gifre et al., 2013; Rossini et al., 2013; Guañabens et al., 2014). Wnt signaling activation through adenosine A_2A_ receptor stimulation has never been described, at least in bone; however, it was already known that A_2B_ ligands stimulates osteogenesis (Ciciarello et al., 2013). Our results demonstrate an involvement of both the canonical and non-canonical Wnt pathways through the activation of 10b and 5a by PDRN. For the first time, we provided “*in vivo*” experimental evidence that activation of the adenosine receptors-mediated signaling exerts anti-osteoporotic effects; even so, our study is affected by the lack of a deeper exploitation of the Wnt pathway involvement in PDRN-driven observed effects, as well as of a better quantification of PDRN-mediated osteoblast differentiation. On the other hand, the drug used in this study could be readily available for clinical trials, because is already used, with good results, in the clinical setting (Squadrito et al., 2014). Overall, if confirmed by additional and deepest *in vitro*/*in vivo* findings, the here-presented pharmacological strategy may represent a significant step forward in the management of bone loss due to glucocorticoids chronic usage.

## Key Messages

• PDRN counteracts glucocorticoid-induced osteoporosis improving bone formation and bone microarchitecture.
• PDRN acts through the adenosine receptor stimulating osteoblasts likely via the Wnt pathway.
• PDRN might be worth of further investigation as an antiosteoporotic treatment.

## Author Contributions

Study design: AB, GaP, and FS. Study conduct: AB, GaP, NI, FG, and GiP. Data collection: GC, FM, AD, EP, and FA. Data analysis: AB and GaP. Data interpretation: AB, GaP, GO, MA, and FS. Drafting manuscript: AB and GaP. Revising manuscript content: FS, GaP, and DA. Approving final version of manuscript: All authors. AB takes responsibility for the integrity of the data analysis.

## Conflict of Interest Statement

Author FS has received research support from Mastelli for work on polydeoxyribonucleotide. Authors FS, AB and DA are co-inventors on a patent describing therapeutic polydeoxyribonucleotide activity in chronic intestinal disease. The remaining authors declare that the research was conducted in the absence of any commercial or financial relationships that could be construed as a potential conflict of interest.

## Footnotes

1
http://rsb.info.nih.gov/ij/
