# Corrigendum: Adenosine receptor stimulation improves glucocorticoid-induced osteoporosis in a rat model

**DOI:** 10.3389/fphar.2022.1073618

**Published:** 2022-11-02

**Authors:** Gabriele Pizzino, Natasha Irrera, Federica Galfo, Giacomo Oteri, Marco Atteritano, Giovanni Pallio, Federica Mannino, Angelica D’Amore, Enrica Pellegrino, Federica Aliquò, Giuseppe P. Anastasi, Giuseppina Cutroneo, Francesco Squadrito, Domenica Altavilla, Alessandra Bitto

**Affiliations:** ^1^ Department of Clinical and Experimental Medicine, University of Messina, Messina, Italy; ^2^ Department of Biomedical Sciences, Dentistry, Morphological and Functional Images, University of Messina, Messina, Italy

**Keywords:** PDRN, Adenosine, glucocorticoids, osteoporosis, rats

In the published article, the **Conflict of Interest** statement was incorrectly filed. The correct Conflict of Interest statement appears below.

“Author FS has received research support from Mastelli for work on polydeoxyribonucleotide. Authors FS, AB and DA are co‐inventors on a patent describing therapeutic polydeoxyribonucleotide activity in chronic intestinal disease. The remaining authors declare that the research was conducted in the absence of any commercial or financial relationships that could be construed as a potential conflict of interest.”

The authors apologize for this error and state that this does not change the scientific conclusions of the article in any way. The original article has been updated.

